# Early life stress during the neonatal period alters social play and Line1 during the juvenile stage of development

**DOI:** 10.1038/s41598-021-82953-3

**Published:** 2021-02-11

**Authors:** Amelia Cuarenta, Stacey L. Kigar, Ian C. Henion, Liza Chang, Vaishali P. Bakshi, Anthony P. Auger

**Affiliations:** 1 Department of Psychology, University of Wisconsin-Madison, Madison, USA; 2 Molecular and Cellular Pharmacology Training Program, University of Wisconsin-Madison, Madison, USA; 3 Department of Psychiatry, University of Wisconsin-Madison, Madison, USA; 4 Neuroscience Training Program, University of Wisconsin-Madison, Madison, USA

**Keywords:** Social behaviour, Sexual dimorphism

## Abstract

Early life stress (ELS) has been shown to have a significant impact on typical brain development and the manifestation of psychological disorders through epigenetic modifications that alter gene expression. Line1, a retrotransposon associated with genetic diversity, has been linked with various psychological disorders that are associated with ELS. Our previous work demonstrated altered Line1 DNA copy number in the neonatal period following stressful experiences; we therefore chose to investigate whether early life stress altered Line1 retrotransposition persists into the juvenile period of development. Our study uses a neonatal predator odor exposure (POE) paradigm to model ELS in rats. We examined Line1 using qPCR to assess Line1 expression levels and DNA copy number in the male and female juvenile amygdala, hippocampus and prefrontal cortex—areas chosen for their association with affective disorders and stress. We report a sex difference in Line1 levels within the juvenile amygdala. We also find that ELS significantly increases Line1 DNA copy number within the juvenile amygdala which correlates with reduced juvenile social play levels, suggesting the possibility that Line1 may influence juvenile social development.

## Introduction

Events experienced early in life can have lasting consequences for brain development and behavior. A plethora of research suggests adverse experiences during early childhood increase the likelihood of experiencing a variety of psychological disorders later in life^1–4^ such as anxiety and depression^1,2,5,6^; therefore, it is profoundly important to understand mechanistically how early-life adversity has a lasting impact on both brain development and risk for psychological disorders.

Early life experiences can manifest via epigenetic modifications that can be either transient or long-lasting^7,8^. Epigenetics refers to changes in DNA expression that do not result in changes to the DNA sequence^9–11^ and are generally thought to occur through modifications made to the DNA itself via DNA methylation or through alterations made to histones^7,8^. Ultimately, these changes have the capacity to alter gene expression levels, in turn impacting behavior, disease susceptibility, and life trajectory. An emerging concept by which the environment can modify DNA is via altered retrotransposon activity^12,13^ suggesting a possible mechanism by which experiences can induce variations in the genome sequence.

Retrotransposons are autonomous elements that are active in a variety of species^12,14^ and are capable of self-replication^14,15^. Long interspersed element 1 (Line1) appears to be the only retrotransposon both present and active in humans, non-human primates, and rodents^14,15^. Line1—comprised of a promoter, two open reading frames (Orfs) and a polyA tail^16,17^—is a 6-kb, autonomous element capable of self-replication and reintegration within the genome^14,17^. Open reading frame 1 protein (Orf1p) codes for a nucleic acid chaperone protein while open reading frame 2 protein (Orf2p) codes for a reverse transcriptase and an endonuclease, both of which assist in reintegration of the element back into the genome^14,15,18^.

Once integrated into the genome, the new Line1 insertion has the potential to disrupt or alter gene expression. Incorporation of both open reading frames appear necessary for newly integrated Line1 elements to have the capacity to be retrotranspositionally active;’ however, the majority of Line1 retrotransposons insertions are 5′ truncated and thus retrotranspositionally inactive^15,19^. Still, truncated Line1 genomic insertions can alter our genetic code by inducing exon-skipping or altering splicing patterns and this in turn can have significant consequences on the normal functioning of genes^20–22^. Furthermore, Line1-mediated retrotransposition events have been classified as mutagenic in a number of instances because of their location of insertion^16^. The functional consequence of insertion into non-coding regions, thereby increasing genetic material, remains to be determined. Therefore, it is important to determine whether environmental factors impact Line1 mobilization. Considering Line1 makes up ~ 17% and ~ 24% of the human and rat genomes, respectively^23^, we believe more research into Line-1 mobilization and expression is warranted.

Indeed, alterations in Line1 DNA copy number have been associated with a number of disorders, including depression, schizophrenia, and bipolar disorder^20–22^, all of which can manifest with atypical social behavior^24–26^. We recently reported that early life stress induces Line1 mobilization and thereby genetic diversity within the neonatal brain^13^. Specifically, we found that early life stress increased Line1 copy number in the neonatal male hippocampus. As in our previous work, our model uses neonatal predator odor exposure (POE) to understand how adverse early life events impact the development of the juvenile brain^27,28^. Previous studies have reported that predator odor induces a stress response in neonates and results in lasting changes within the brain and on behavior^13,27,29–33^. Animals were exposed to POE on postnatal days 1, 2 and 3 and raised to the juvenile time period to assess whether their early life experience produced lasting changes to Line1 activity and social behavior. We used juvenile social play as an indicator of typical social development. Disruption of juvenile social play can hinder brain and behavior development in many species including humans, non-human primates and rodents. For example, deprivation of juvenile social play in rodents can lead to atypical patterns of social and aggressive behavior during adulthood life^34,35^ while in humans social withdrawal during childhood can lead to dysregulation of emotional responses later in life and increased susceptibility to some psychiatric disorders^36,37^. Therefore, we assess whether juvenile social play was disrupted by early life stress. We examined Line1 activity in the amygdala, hippocampus and prelimbic area commonly referred to as the prefrontal cortex, as these areas are known to be influenced by stress.

## Results

### Juvenile social play behavior

**Figure 1:**
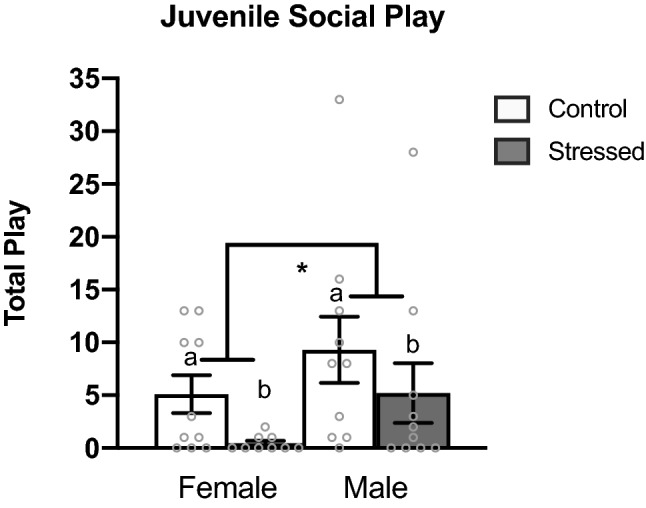
Juvenile social play behavior. Early life stress reduces juvenile social play behavior in males and females. Juvenile males play at higher levels than juvenile females. Values shown as mean ± SEM. *Indicates p < 0.05.

As reported in previous literature^35,38–40^, we found a significant sex difference in total juvenile social play levels with males exhibiting higher levels of social play than females (*p* = 0.043) (Fig. 1). We also found a significant effect of stress, wherein animals that experienced early life stress played at lower levels than control animals (*p* = 0.017) (Fig. 1).

### Relative Line1 DNA copy number

**Figure 2:**
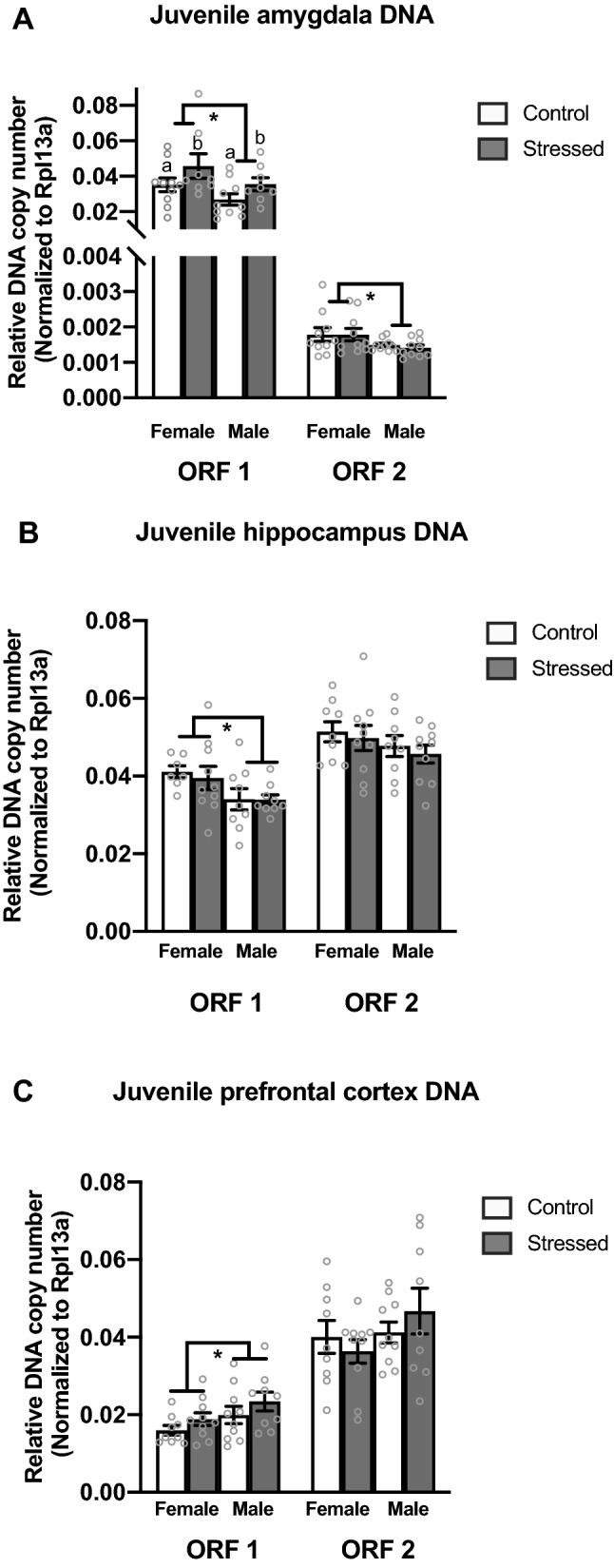
RT-qPCR analysis of Line1 Orf1 and Orf2 mRNA in the juvenile amygdala, hippocampus, and prefrontal cortex. (**A**) Females have more Line1 Orf1 and Orf2 copy number in the amygdala than males. (**B**) Females have more Line1 Orf1 copy number in the hippocampus than males. (**C**) Males have more Line1 Orf1 copy number in the prefrontal cortex than females. Values shown as mean ± SEM. *Indicates p < 0.05.

There was a significant main effect of stress in the juvenile amygdala with stressed animals having more Line1 Orf1 DNA copy number than control animals (*F*_(1,35)_ = 4.505, *p* = 0.042) (Fig. 2A). We also found a significant sex difference in Line1 Orf1 DNA copy number; females have more Line1 Orf1 DNA copy number than males (*F*_1(35)_ = 5.223, *p* = 0.029) (Fig. 2A). We found a significant sex difference in Line1 Orf2 copy number in the juvenile amygdala with females having more Line1 Orf2 copy number than males (*F*_(1,39)_ = 5.714, *p* = 0.022) (Fig. 2A).

In the juvenile hippocampus, there was a significant sex effect in Line1 Orf1 relative DNA copy number with males having fewer copies than females (*F*_(1,34)_ = 6.84, *p* = 0.014) (Fig. 2B). There was no significant difference in relative copy number in Line1 Orf2 in the hippocampus (Fig. 2B). Lastly, in the juvenile prefrontal cortex we identified a significant sex effect in Line1 Orf1 with males having higher relative genomic copy number than females (*F*_(1,37)_ = 4.729, *p* = 0.037) (Fig. 2C). There was no significant difference in Line1 Orf2 relative copy number in the prefrontal cortex (Fig. 2C).

### Line1 mRNA levels

**Figure 3:**
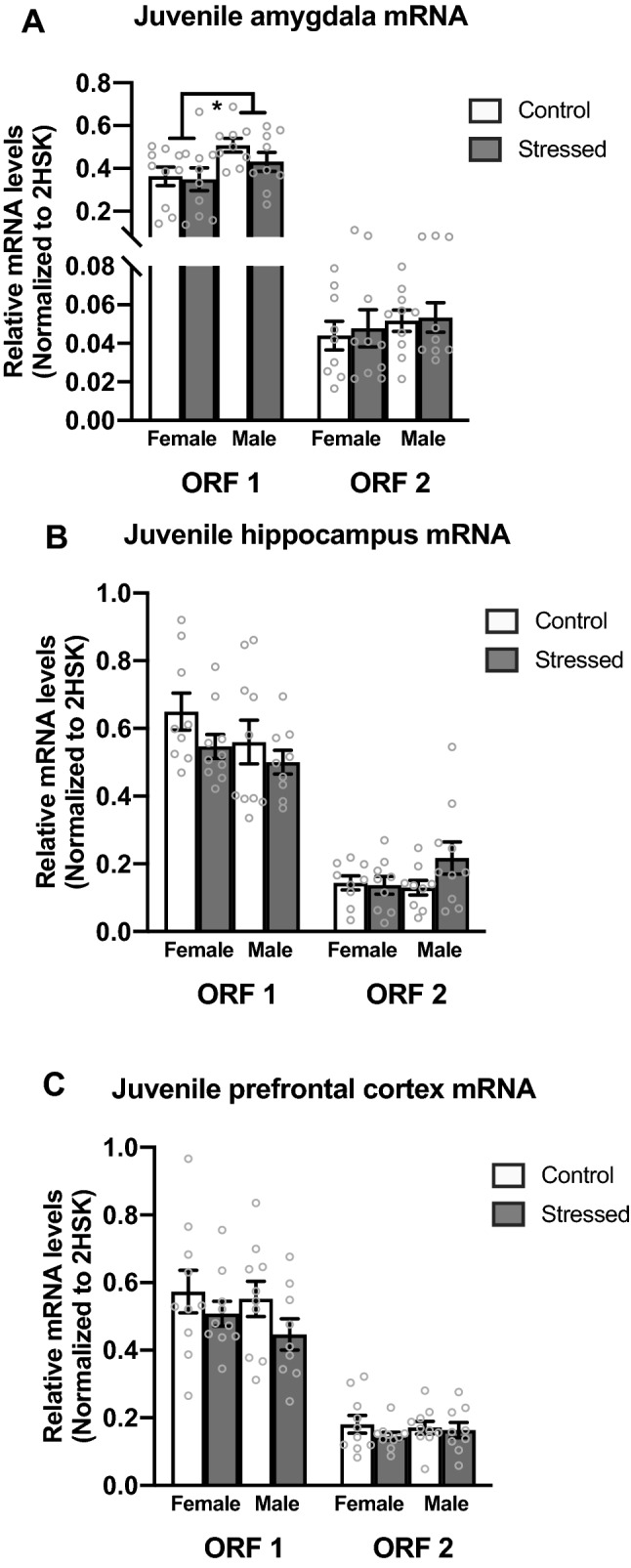
RT-qPCR analysis of Line1 Orf1 and Orf2 mRNA in the juvenile amygdala, hippocampus, and prefrontal cortex. (**A**) Males have greater Line1 mRNA expression than females in the juvenile amygdala. (**B**) No changes in Line1 mRNA expression were observed in the juvenile hippocampus. (**C**) No changes in Line1 mRNA expression were observed in the juvenile prefrontal cortex. Values shown as mean ± SEM. *Indicates p < 0.05.

There was a significant sex difference in Line1 Orf1 mRNA expression within the amygdala. Males showed higher Line1 mRNA expression levels than females (*F*_(1,37)_ = 6.484, *p* = 0.016) (Fig. 3A). There was no significant difference in mRNA expression in Line1 Orf2 in the amygdala (Fig. 3A). There was no significant difference in either Line1 Orf1 or Orf2 mRNA levels within the juvenile hippocampus (Fig. 3B) or prefrontal cortex (Fig. 3C).

### Juvenile social play behavior correlates with Line1

**Figure 4:**
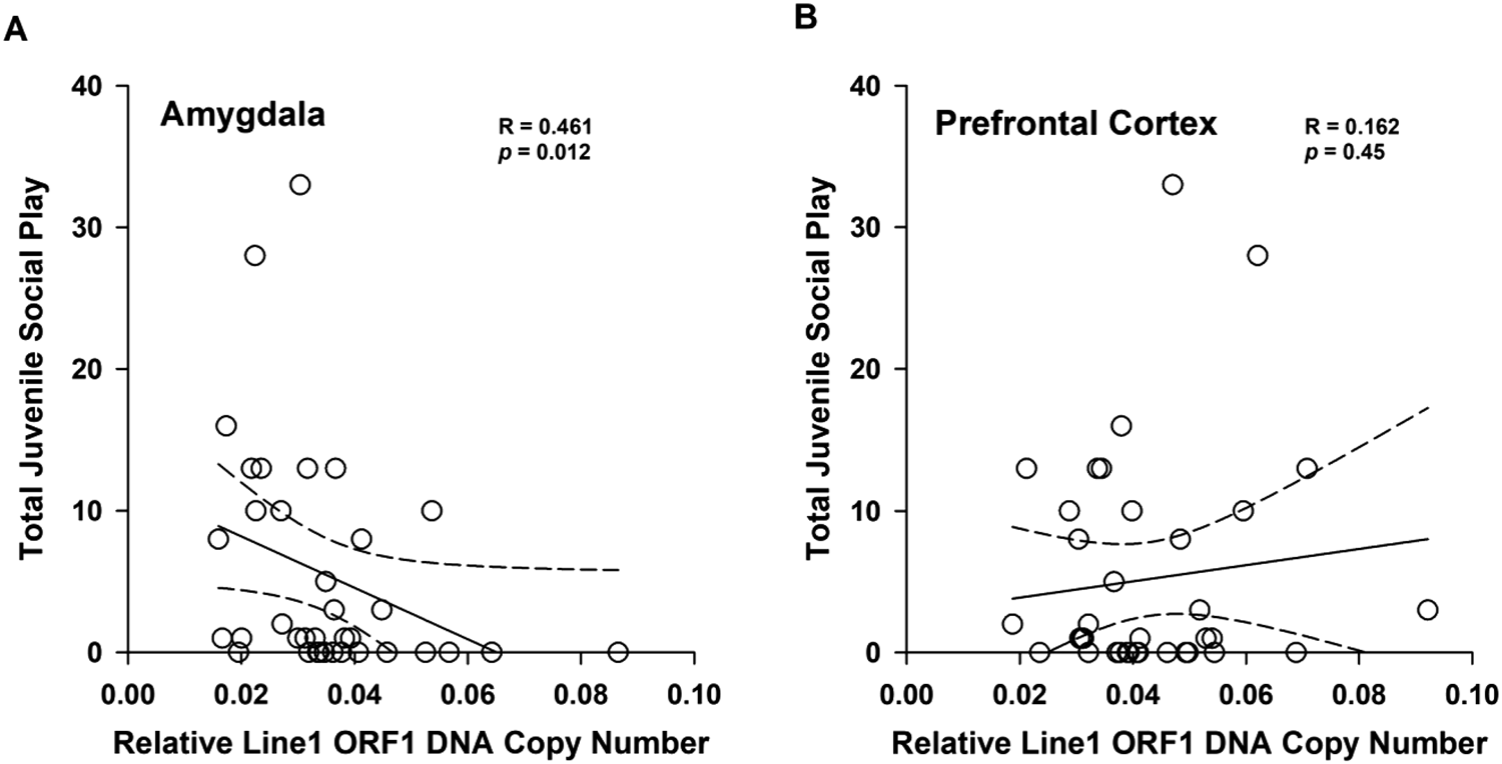
(**A**) Juvenile social play levels correlate with relative Line1 Orf1 DNA copy number in the amygdala. (**B**) Juvenile social play levels are not correlated with relative Line1 Orf1 DNA copy number in the prefrontal cortex.

We used a linear regression analysis to examine whether differences in Line1 DNA copy number within the amygdala correlated with the levels of juvenile social play. We found that Line1 Orf1 DNA copy number in the amygdala correlated with juvenile social play levels r(34) = 0.416, (*p* = 0.012). Animals with higher Line1 Orf1 DNA copy number played at lower levels (Fig. 4). We also examined the prefrontal cortex and did not find a correlation between Line1 copy number and juvenile social play.

## Discussion

We sought to examine whether juvenile social play and Line1 DNA copy number differed as a result of early life stress and/or biological sex in the juvenile brain. Our previous findings indicated that early life stress resulted in increased Line1 DNA copy number in the male hippocampus within a few days following stress^13^; however, our current data suggests that these immediate stress-induced changes were no longer detected within the juvenile hippocampus. In contrast, exposure to early life stress altered Line1 activity within the juvenile amygdala, a region critical for juvenile social play. Furthermore, our data suggest that there are sex differences in Line1 DNA copy number within the juvenile brain and that these sex differences are region-specific. This suggests that altered retrotransposon activity during development adds an additional layer of complexity in understanding how sex differences are manifested in the brain and how early adverse experiences impact the genome in a lasting manner.

One of the more frequent social behaviors to occur during the juvenile period is social play. Juvenile social play is a complex behavior that is highly sensitive to prior early life experiences. Numerous labs, including our own, have reported sex differences in the levels of juvenile social play, with males engaging in higher levels of rough-and-tumble play behavior than females^38–43^. Therefore, we examined whether our paradigm of ELS would negatively impact later juvenile social play. As expected, sex differences were found in total juvenile social play behavior with males exhibiting higher play levels than females (Fig. 1). Interestingly, we also found that ELS disrupted juvenile social play; animals that experienced POE played at significantly lower levels than control animals (Fig. 1). Prior work has found that lipopolysaccharide injections reduced juvenile social play levels in males^44^. These data indicate that stressors early in life can lead to disengagement of juvenile social play.

As variations of Line1 copy number have been associated with disorders that present with altered social behavior, we then examined how ELS impacted the levels of Line1 DNA copy number within the juvenile brain. Within the juvenile amygdala, we found a significant difference in Line1 DNA copy number among treatment groups; specifically, stressed animals had more Line1 Orf1 DNA copy number than control animals in the juvenile amygdala (Fig. 2A). We also found significant sex differences in Line1 DNA copy number (Fig. 2A) in which females have higher Line1 DNA copy levels in the juvenile amygdala. This pattern holds true for both open reading frames (Fig. 2A). Given that the amygdala is involved in juvenile social play^38,45^, we wanted to examine whether the disruption in juvenile social play correlated with Line1 DNA copy number in the amygdala. A significant negative correlation between Line1 Orf1 DNA copy number within the amygdala and juvenile social play behavior was found; animals with more Line1 Orf1 DNA copy number played at lower levels (Fig. 4). These data suggest that exposure to stressful events early in life may result in the mobilization of Line1 and decreases in juvenile social interactions, such as social play, which are usually thought to confer a positive effect on development^38,46,47^. Therefore, the POE-induced disruption in juvenile social play may indirectly impair adult social development.

While we previously reported that exposure to a variable predator odor paradigm on postnatal days 1–3 resulted in an increase in Line1 Orf1 DNA copy number in the neonatal hippocampus in males within a few days^13^, we found no lasting differences in Line1 DNA copy within this region. These findings could reflect altered Line1 retrotransposon mobilization and stability within the genome due to cell turnover, or cell migration across hippocampal development, or some other interference with the detection of Line1^48–50^. While there are numerous DNA repair enzymes that can correct DNA damage or alterations, it remains to be elucidated if repair enzymes exist to remove Line1 sequences from the genome.

Our current study also finds intriguing variations in the levels of Line1 DNA copy number influenced by biological sex. Within the juvenile hippocampus, females have higher DNA levels of Line1 Orf1 than males (Fig. 2B). In contrast, we find the opposite in the prefrontal cortex. That is, within the juvenile prefrontal cortex, we found that males had significantly higher Line1 Orf1 DNA levels than females (Fig. 2C). These data suggest that not only do we have genetic mosaicism within the developing brain, but it is occurring in a sex- and regionally-specific pattern. This further implies an individual’s somatic cells are not genetically identical, lending additional support to the notion of genetic mosaicism established by others^14,51–53^. This genomic variability between cell types may allow for a more dynamic response to environmental experiences and perhaps demonstrates a unique plasticity within our genome via alterations to the underlying DNA sequence itself in response to environmental challenges during development. To determine a more proximal cause of Line1 mobilization, it would be interesting to examine how treatment with gonadal hormones or stress hormones alters Line1 mobilization patterns in the developing brain. These additional studies will help elucidate whether Line1 insertions are random or if there is a targeted approach of insertion to specific brain regions or loci.

Within the juvenile amygdala, we observe females have more Line1 Orf1 and Orf2 copy number than males. Line1 Orf1 encodes for a nucleic acid chaperone protein that is necessary for retrotransposition^14,18,54,55^ while Line1 Orf2 encodes both an endonuclease and a reverse transcriptase^14,15^. Both open reading frames are necessary for successful retrotransposition, but the large majority of insertions only include a small portion of the entire sequence, rendering them retrotranspositionally incompetent^15,19^. As females have higher levels of both Orf1 and Orf2 DNA copy number in the amygdala, these data suggest that the novel Line1 insertions within the female juvenile amygdala have the capacity to mobilize again in response to environmental stimuli.

In contrast to the juvenile amygdala, we find sex differences in only Line1 Orf1 within the hippocampus and prefrontal cortex. Therefore, it is likely that the increase in Orf1 DNA copy number in the female hippocampus and the increase in Orf1 in the male prefrontal cortex will not yield new “hotspots” that are capable of active retrotransposition. However, the incorporation of single open reading frames can have profound effects on the genome even if not retrotranspositionally active. The location of Line1 insertions can alter the expression of other genes if inserted into promoter or coding regions of genes^56^. Line1 insertions can create missense or nonsense mutations^15,57^ or can alter gene expression by changing splicing patterns or inducing exon skipping^15,58,59^. To better understand the functional consequence of Line1 mobilization, it will be important to understand where Line1 insertions are occurring within the genome. While this remains to be determined, some research suggests that retrotransposon insertions may be biased towards AT rich regions^60^. Further work determining the exact location of Line1 incorporation during development is warranted.

We also sought to understand whether mRNA levels would be altered by ELS or biological sex in the juvenile brain. While we did not find any evidence that ELS altered mRNA levels in the juvenile period, we did find a significant sex difference in Line1 mRNA levels. Within the juvenile amygdala, we found that males had increased Line1 Orf1 mRNA expression compared to females (Fig. 3A). There were no differences within Orf2. We found no significant differences in mRNA levels of either Line1 Orf1 or Orf2 mRNA levels within the juvenile hippocampus (Fig. 3B) or the prefrontal cortex (Fig. 3C). The increase in RNA in the male amygdala is likely to be retrotranspositionally inactive, as both reading frames are needed to produce a retrotranspositionally competent locus. It is important to note that the proteins produced by Line1 mRNA are not only for Line1 incorporation but can also be used by another retrotransposon, short interspersed nuclear element (SINE), for its insertion into the genome^14,15^. Future research should explore the functional consequence of increased mRNA on SINE incorporation.

Collectively, our data demonstrate that adverse experiences early in life (i.e., exposure to predator odor) can create enduring alterations in Line1 levels within the juvenile brain. This suggests that stressful experiences early in life have the capacity to change the genome via altered retrotransposon activity. Furthermore, these Line1 genomic changes within the amygdala may be linked to disrupted juvenile social play behavior. It is important to note that different stressors may field different responses. For example, early life stress associated with maternal separation can increase levels of juvenile social play^61^, whereas early life stress from lipopolysaccharide injections can decrease the levels of juvenile social play^44^. Additionally, exposure to the same stressor at different developmental time periods can produce different neuroendocrine and gene expression responses within the brain^62^. Given that we explored stress during the neonatal hyporesponsive period, these effects would likely be different if stress were experienced at a different developmental timepoint. Indeed, since juveniles are more responsive to stress^63^ it is possible we would see greater effects if POE were applied during this time. Further research exploring developmental timing and different stress models is warranted to assess if they induce different patterns of Line1 mobilization. Our data also support the possibility that somatic mosaicism occurs within the developing brain and that this pattern is sex specific. Therefore, it is important to continue to examine Line1 retrotranspositional events with biological sex as a variable of interest. It is necessary to understand not only where Line1 insertions are being incorporated within the genome, but also what the functional consequence of Line1 insertions have within the developing brain and on social development.

## Methods

### Animals

Untimed pregnant Sprague–Dawley dams were purchased from Charles River Laboratories (Wilmington, MA/Charles River, Kingston, NY) and kept in the Wisconsin Psychiatric Institute and Clinical animal facilities. Animals were allowed to deliver naturally, and cages were regularly checked to determine the day of birth (Postnatal day 0, P0). On P0 approximately 6 h post-birth, litters were culled to a composition of 5 females and 5 males per litter (n = 10) with litters pooled and randomized. Animals underwent the POE from P1 to P3 and then were allowed to develop undisturbed until weaning on P21 at which time animals were housed in mixed-sex littermate cages (n = 5 animals per cage). Animals were sacrificed on P33. All animals were housed under standard laboratory conditions (reverse light/dark cycle of 12 h/12 h, food and water ad libitum). All experiments were performed in accordance with the ARRIVE guidelines^64^, NIH Guide for Care and Use of Laboratory Animals in Research, and all procedures were approved by the University of Wisconsin-Madison Animal Care and Use Committee.

### Predator odor exposure (POE)

As we previously reported^27,28^ predator odor exposure (POE) was used to model early life stress (ELS). This paradigm was adapted to neonates from a well-established paradigm of predator stress for adult Sprague–Dawley rats^4,65,66^ to model the effects of early life adversity in the juvenile brain. Prior work demonstrates that predator odor elicits a response by neonates during the hyposensitive period^29^. Our paradigm was designed to reduce potential odor habituation effects by using three different odors. Pups were exposed to the predator odors of cat, rat, and ferret on postnatal days 1, 2, and 3 respectively for 5 min each day. Animals were removed from their dam, brought to a separate room and placed into a chamber with no direct contact to the odor. Control animals were handled in the exact same manner but were exposed to clean bedding instead of a predator odor. All animals were returned to their dam within 10 min. Each group (4 groups total) consisted of n = 10 animals per group with at least three litters represented.

### Home cage behavior

Juvenile social play behavior was observed directly in the home cage, and scoring was adapted from previously reported methods^41,67^. All animals in the homecage were part of the experiment and there were 4 homecages for each group analyzed. Animals were marked on their backs and tails for identification using a non-toxic permanent marker 2-h before the beginning of the dark phase of the light cycle on postnatal days P25–P30. Home cage behavior was video-recorded for 5 min each day from P25–P30 for a total observation time of 25 min per animal. All videos were analyzed and scored by trained observers blind to biological sex and condition using The Observer software (Noldus Information Technologies).

Juvenile social play was analyzed and scored by the following criteria: (1) biting: one rat biting another; (2) boxing: both rats stand on hind legs and engage each other with forepaws; (3) chasing: one rat chases another; (4) pinning: one rat standing over another with its forepaws on the ventral surface of the opposing rat; and (5) pouncing: one rat pounces or lunges at another^38,68^. Individual behaviors were observed and recorded (Supplementary Table S1). Total juvenile social play is the sum of all components of play behavior over the 5 days.

### Tissue collection

All animals were sacrificed via rapid decapitation. The amygdala, hippocampus, and prefrontal cortex were dissected out and immediately snap-frozen in isopentane (Fisher; catalog #O3551-4) on dry ice. Tissue samples were randomized and then homogenized with total RNA and DNA collected using AllPrep DNA/RNA Mini Kit (Qiagen; catalog #80204)^69^.

### Quantification of mRNA

As previously reported, mRNA concentrations and purity were determined using the NanoDrop 2000 (ThermoScientific; catalog #ND-2000LAPTOP)^27,28^. Messenger RNA was converted to cDNA with the Promega ImProm-II™ Reverse Transcriptase System (Promega; catalog #A3800). Real-time quantitative polymerase chain reaction (qPCR) was conducted using a Stratagene Mx3000PTM real-time PCR system, and cDNA was amplified with Gotaq Colorless Master Mix (Promega; catalog #M7132), SYBR green (Invitrogen; catalog #S33102) and ROX as a reference dye (Invitrogen; 12223-012). Following amplification, dissociation melt curves and DNA gel analyses were performed to ensure purity of PCR products. Relative cDNA levels were calculated using the comparative C_T_ method normalized to the average of two reference genes (Rpl13a and Ywhaz) that showed no statistically significant differences between any groups. Line1 Orf1 and Line1 Orf2 along with all other primers are located in Table 1.

**Table 1:** mRNA and DNA primer sequences for qPCR.

Primer	Method	Forward	Reverse
Line1 Orf 1	qPCR mRNA	GAACCCAAGCAACAGAAACCA	CCATGTTTGTTTTGGCGGGA
Line1 Orf 2	qPCR mRNA	TCTATGCCCCAAATACAAG	AGTTTTCCTCTTAGCACAGC
Rpl13a	qPCR mRNA	AGCAGCTCTTGAGGCTAAGG	GGGTTCACACCAAGAGTCCA
Ywhaz	qPCR mRNA	TTGAGCAGAAGACGGAAGGT	GAAGCATTGGGGATCAAGAA
Line1 Orf 1	qPCR DNA	GAACCCAAGCAACAGAAACCA	CCATGTTTGTTTTGGCGGGA
Line1 Orf 2	qPCR DNA	GTGCGATTGGCTAAGATC	GAGTGTTCCTCTTTCTCCACAACCT
Rpl13a	qPCR DNA	ATGAACCCCAAGTAAGCAGGG	ATAGGCATCCTTGTGGGGAGA

### Quantification of DNA

DNA concentrations and purity were determined using the NanoDrop 2000 (ThermoScientific; catalog #ND-2000LAPTOP) as previously reported^13,27,28^. Real-time quantitative polymerase chain reaction (qPCR) was conducted using a Stratagene Mx3000PTM real-time PCR system, and DNA amplified with Gotaq Colorless Master Mix (Promega; catalog #M7132), SYBR green (Invitrogen; catalog #S33102) and ROX as a reference dye (Invitrogen; 12223-012). Following amplification, dissociation melt curves and DNA gel analyses were performed to ensure purity of PCR products. Relative DNA levels were calculated using the comparative C_T_ method normalized to the reference gene Rpl13a which showed no statistically significant difference between any group. All primers are located in Table 1.

### Statistical analysis

All molecular tests were conducted and analyzed by researchers unaware of group classification. All PCR data passed normality and were analyzed with two-way ANOVAs and Tukey post-hoc for each gene and each brain region. Due to variations in the levels of social play observed over the 5 days, normality was not met, so data were square root transformed. Statistical analyses were performed using SigmaPlot 11.0. Potential outliers were screened for using the Grubbs test for outliers (http://graphpad.com/quickcalcs/ Grubbs1.cfm). All reported measures are listed as mean ± SEM. Significance is defined as a *p*-value of < 0.05.

## Supplementary Information

Supplementary Information.
