# Supplementary material for: Early life stress during the neonatal period alters social play and Line1 during the juvenile stage of development
Source: Sci Rep. 2021 Feb 11;11:3549. doi: 10.1038/s41598-021-82953-3 (PMC7878767; doi:10.1038/s41598-021-82953-3)
Supplement: Supplementary file 1 — Supplementary Information. [file 41598_2021_82953_MOESM1_ESM.pdf]

Early life stress during the neonatal period alters social play and Line1 during the juvenile stage of development

Amelia Cuarenta[a], Stacey L. Kigar[b], Ian C. Henion[a], Liza Chang[a], Vaishali P. Bakshi[c], Anthony P. Auger[a,d]

[a]Department of Psychology, University of Wisconsin-Madison
[b]Molecular and Cellular Pharmacology Training Program, University of Wisconsin-Madison
[c]Department of Psychiatry, University of Wisconsin-Madison
[d]Neuroscience Training Program, University of Wisconsin-Madison

Amelia Cuarenta — cuarenta@wisc.edu
*Anthony Auger (corresponding author) apauger@wisc.edu

Supplemental Figures

Table S1. Components of Juvenile Social Play Behavior

	Bite	Box [A]	Chase	Pounce	Pin [B]
**Control Female**	Mean= 0<br>SE= 0	Mean= 0.7<br>SE= 0.21	Mean= 0.1<br>SE= 0.1	Mean= 2<br>SE= 0.82	Mean= 2.3<br>SE= 0.99
**Stressed Female**	Mean= 0<br>SE= 0	Mean= 0.12<br>SE= 0.12	Mean= 0<br>SE= 0	Mean= 0.34<br>SE= 0.17	Mean= 0<br>SE= 0
**Control Male**	Mean= 0<br>SE= 0	Mean=1.4<br>SE= 0.34	Mean= 0<br>SE= 0	Mean= 3.9<br>SE= 1.43	Mean= 4<br>SE= 1.61
**Stress Male**	Mean= 0.3<br>SE=0.3	Mean= 0.7<br>SE= 0.26	Mean= 0.3<br>SE= 0.21	Mean= 2.6<br>SE= 2.06	Mean= 1.3<br>SE= 0.56

Table indicates mean frequency of individual behavioral components that make up total play. No differences were found in chase, bite, or pounce. There were main effects of sex and POE on boxing behavior[A], indicating that males engaged in more boxing behavior than females ($p=0.021$) and that POE reduces boxing behavior in males and females ($p=0.01$). Additionally, while there was a trend for a sex difference in pinning behavior, it did not reach statistical significance ($p=0.06$); however, there was a main effect of POE on reducing overall levels of pinning behavior[B] ($p=0.013$).
